# A Novel Candidate Region for Genetic Adaptation to High Altitude in Andean Populations

**DOI:** 10.1371/journal.pone.0125444

**Published:** 2015-05-11

**Authors:** Guido Valverde, Hang Zhou, Sebastian Lippold, Cesare de Filippo, Kun Tang, David López Herráez, Jing Li, Mark Stoneking

**Affiliations:** 1 Australian Centre for Ancient DNA, School of Earth & Environmental Sciences, The University of Adelaide, Adelaide, Australia; 2 Department of Computational Regulatory Genomics, CAS-MPG Partner Institute for Computational Biology, Shanghai Institutes for Biological Sciences, Shanghai, China; 3 Department of Evolutionary Genetics, Max Planck Institute for Evolutionary Anthropology, Leipzig, Germany; 4 Department Effect-Directed Analysis, Helmholtz Centre for Environmental Research—UFZ, Leipzig, Germany; Kunming Institute of Zoology, Chinese Academy of Sciences, CHINA

## Abstract

Humans living at high altitude (≥2,500 meters above sea level) have acquired unique abilities to survive the associated extreme environmental conditions, including hypoxia, cold temperature, limited food availability and high levels of free radicals and oxidants. Long-term inhabitants of the most elevated regions of the world have undergone extensive physiological and/or genetic changes, particularly in the regulation of respiration and circulation, when compared to lowland populations. Genome scans have identified candidate genes involved in altitude adaption in the Tibetan Plateau and the Ethiopian highlands, in contrast to populations from the Andes, which have not been as intensively investigated. In the present study, we focused on three indigenous populations from Bolivia: two groups of Andean natives, Aymara and Quechua, and the low-altitude control group of Guarani from the Gran Chaco lowlands. Using pooled samples, we identified a number of SNPs exhibiting large allele frequency differences over 900,000 genotyped SNPs. A region in chromosome 10 (within the cytogenetic bands q22.3 and q23.1) was significantly differentiated between highland and lowland groups. We resequenced ~1.5 Mb surrounding the candidate region and identified strong signals of positive selection in the highland populations. A composite of multiple signals like test localized the signal to *FAM213A* and a related enhancer; the product of this gene acts as an antioxidant to lower oxidative stress and may help to maintain bone mass. The results suggest that positive selection on the enhancer might increase the expression of this antioxidant, and thereby prevent oxidative damage. In addition, the most significant signal in a relative extended haplotype homozygosity analysis was localized to the *SFTPD* gene, which encodes a surfactant pulmonary-associated protein involved in normal respiration and innate host defense. Our study thus identifies two novel candidate genes and associated pathways that may be involved in high-altitude adaptation in Andean populations.

## Introduction

It is generally accepted that anatomically modern humans emerged in Africa and radiated from there to colonize most of the world's land masses [1]. During this “out of Africa” diaspora, modern humans encountered new habitats with a very diverse set of ecological conditions in contrast to the African homeland, e.g., in the form of new geographic environments, climates, diets and/or pathogens. Humans adapted successfully to these new conditions both culturally and biologically, the latter involving physiological acclimatization and/or genetic adaptation. One of the extreme habitats successfully colonized by humans is high altitude.

The main environmental stresses of living in elevated plateaus and mountainous regions are the decrease of temperature and humidity, the increase in solar electromagnetic radiation, and hypobaric hypoxia (defined as the decrease in oxygen intake for metabolic processes due to reduced barometric pressure) [2,3]. Although the term “high altitude” has no precise definition, it is generally taken to refer to regions that are 2,500–3,000 meters (m) or more above sea level, as the majority of newcomers arriving in such regions present certain clinical, physiological, anatomical and biochemical changes (reviewed in [3]). Moreover, some populations of humans have developed a physique that enables permanent habitation of high-altitude regions despite the severe conditions of hypoxia and other environmental stressors.

Three main high-altitude regions of the world have supported relatively large human populations for millennia: the Ethiopian highlands of the Semien Mountains [4,5], the Tibetan Plateau and Himalayan valleys [6,7], and the Andes of South America [2,8,9]. In order to overcome hypobaric hypoxia, the human body needs to adjust the cascade of metabolic processes for oxygen uptake and utilization. However, there is no universal pattern of response to hypoxia. People living in each of the above mentioned regions exhibit diverse respiratory, circulatory, hematological, and even pathological patterns of acclimatization and/or adaptation. For example, there is a relatively low hemoglobin concentration in Ethiopian and Tibetan highlanders as opposed to Andean or European populations living at high altitude [4,10,11]. Tibetans, similar to sojourners, display higher hypoxic ventilatory response (HVR) which results in increased ventilation compared with Andeans [12,13]. Furthermore, chronic mountain sickness (CMS), a disease defined as loss of adaptation to altitude [14], is common in the Andes, occasionally found in the Himalayas, and absent from the Ethiopian highlands [5,15]. CMS has a strong familial component, and it has been also noted that in Bolivian Andeans CMS is predominant in males of mixed or entirely European genetic background [16,17]. Moreover, having been born and raised within multigenerational high-altitude residence families appears to confer a substantial advantage in survival and performance at high-altitude environments (reviewed in [18]). This is in accordance with expectations that distinctive traits between high and low-altitude natives (or between different high-altitude native groups) may reflect genetic adaptations resulting from natural selection.

The characteristic morphology and physiology of high-altitude natives, in particular Tibetans and Andeans, has been studied in detail [11], enabling the identification of underlying candidate genes or groups of genes (e.g., [19]), such as the hypoxia-inducible transcription (HIF) pathway, renin-angiotensin system (RAS), and nitric oxide synthases (NOSs) [20–26]. However, because of the limited genomic scope of many candidate-gene studies, functionally relevant variation may have been overlooked. Moreover, the use of rather dissimilar lowland population outgroups, for example comparing Andeans with controls of European or indigenous North American genetic background (e.g., [20,21]), lessens statistical power. More recently, studies applying genome-wide scans have independently identified genes whose products participate in the HIF pathway (e.g. *EGLN1* and *EPAS1*), and represent strong targets of selection at high altitude, especially for the Tibetan population [27–32]. Besides the HIF pathway, two genes involved in heart performance (*VEGFB* and *ELTD1*) have also been implicated in the elevated hematocrit characteristic of high-altitude populations in the Andes [33], while new candidate-gene sets have been proposed in Ethiopians as well [34–36].

In the present study, we focused on three indigenous populations from Bolivia, namely two groups of native inhabitants living at high altitude (≥3600 meters above sea level) in the Andes, Aymara and Quechua, and as a control group the Guarani from the Gran Chaco lowlands. Using a genome-scan approach and pooled population samples, we identified a candidate region in chromosome 10 that exhibited significant allele frequency differences between the high- and lowland populations. Targeted resequencing of approximately 1.5 Mb of the region of interest revealed strong signals of positive selection in both Andean groups, suggesting that this genomic region harbors genes for high-altitude adaptation in these populations.

## Results

**Fig 1:**
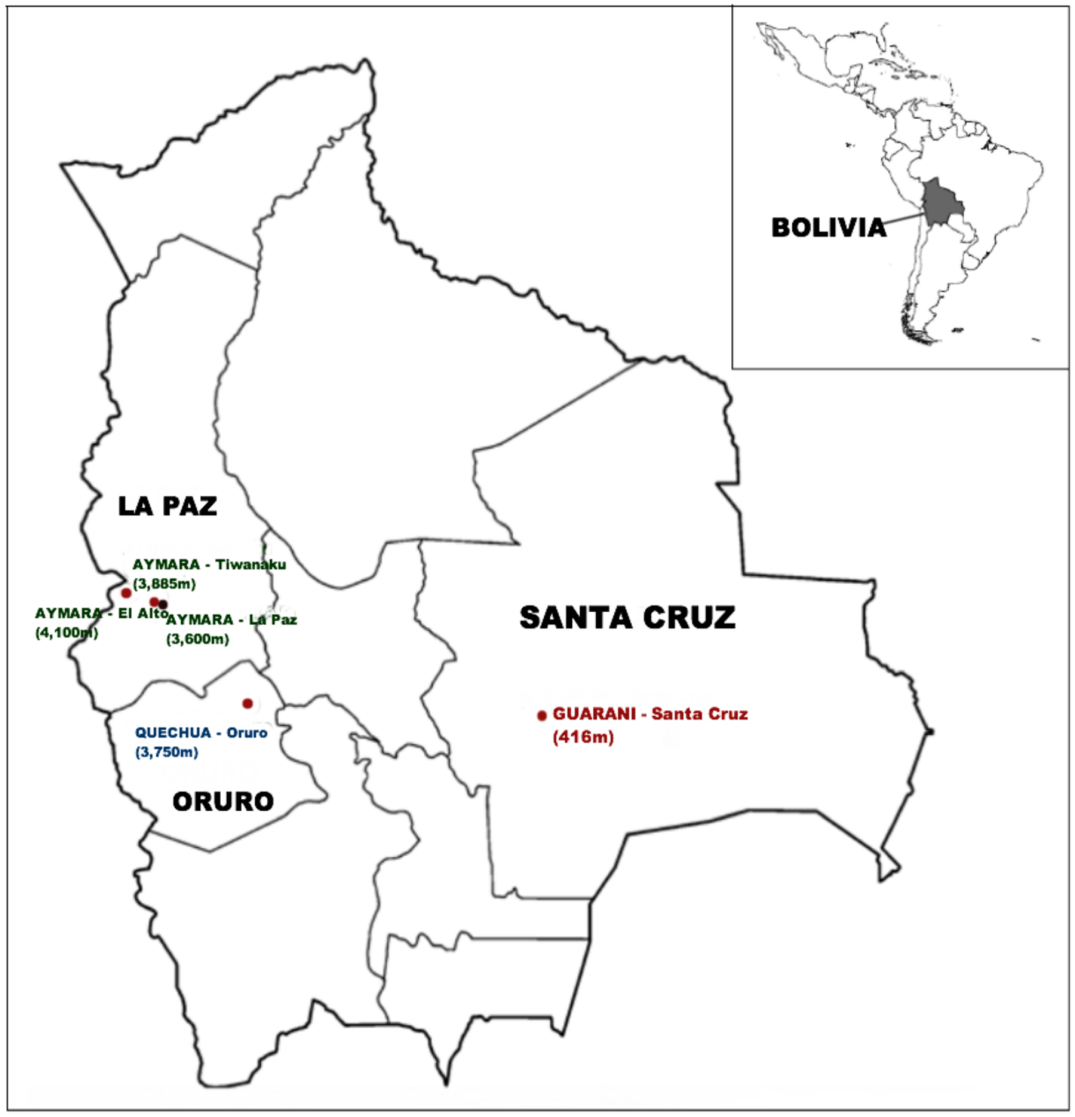
Sampling locations of the three indigenous populations from Bolivia.

We collected saliva samples for the extraction of genomic DNA from 55 (24 males / 31 females) Aymara (AYM) and 21 (18 males / 3 females) Quechua (QUE) from locations above 3,600 meters, and 23 (14 males / 9 females) Guarani (GUA) from the lowlands (see Materials and Methods) in Bolivia (Fig 1). Samples were obtained from healthy individuals with no known medical record or indication of CMS or any other altitude disease. All sampled individuals were unrelated and of self-identified ancestry as Aymara, Quechua or Guarani. Given the high historical rates of post-Columbian colonization male-mediated admixture into Native American communities [37], we performed determination of Y chromosome haplogroups for confirmation of the donor’s ethnicity in all of the male samples. Indeed, the most common haplogroup found in our collection of Bolivian males was Q (predominant branch of the Y phylogeny observed in modern-day Amerindians of Central and South America), at frequencies of roughly 80% in all three groups (S1 Fig).

## Pooled DNA Microarray genotyping and estimation of allele frequencies

To search for highly-differentiated genic regions among the high- and lowland groups, and therefore putatively involved in altitude adaptation, we pooled DNA samples from each population independently in triplicate (see Materials and Methods), genotyped each pool on the Affymetrix Genome-Wide Human SNP Array 6.0, and estimated allele frequency differences between each pair of populations based on the intensity of the hybridization signals. This approach has been used in several studies [38–41]. On average, the SNP call rate was 87.5% for the Aymara pools, 85.1% for the Quechua pools, and 88.2% for the Guarani pools. These rates are comparable to previous results with the Affymetrix platform for pooled DNA (e.g., [39]) or even with DNA from single samples (e.g., [42]).

We used frequency estimates for every called SNP obtained from the microarray experiments to perform pairwise comparisons among the populations, and we identified candidate regions that contained highly differentiated SNPs (see Materials and Methods). The tests were done between all pairs of the three groups as well as between the highlander group (combining Aymara and Quechua, from here on referred to as HL) and the lowlander group (Guarani, from here on LL). Aymara and Quechua share similar environments and lifestyles, and previous studies have found that they are genetically similar as well [33,43,44]. Since the main goal of this study was to investigate loci related to high-altitude adaptation, the following results and discussions will be mainly focused on the HL-LL comparison, as in previous studies [24,32], unless otherwise specified.

**Fig 2:**
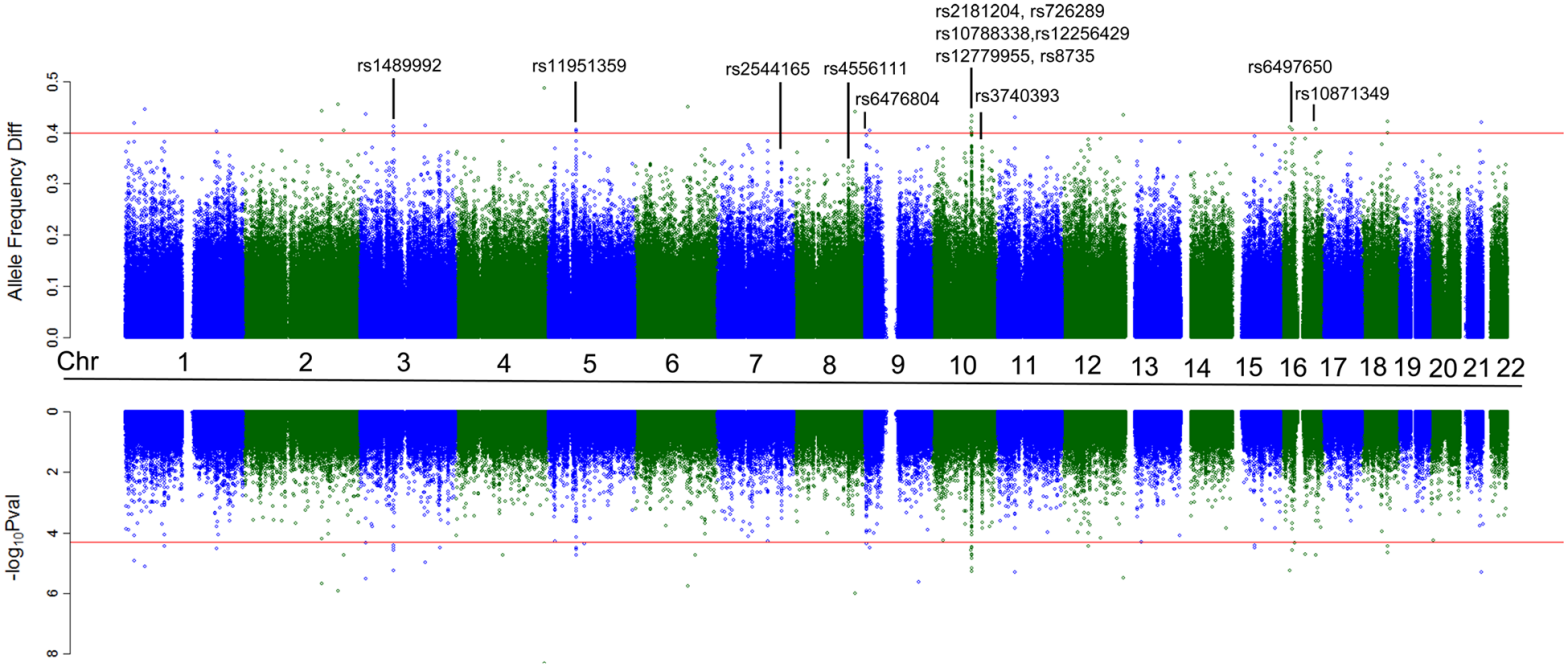
Allele frequency differences between HL and LL revealed by genotyping of pooled DNA samples. The 9 candidate regions and corresponding SNPs exhibiting significant allele frequency differences are labeled. The top red line indicates allele frequency differences of 0.4, and the bottom red line indicates genome-wide significance (P value = 0.5 x 10^–4^).

Based on the HL-LL comparison, we detected 9 candidate regions containing SNPs exhibiting large allele frequency differences (≥ 0.3); these regions were also supported by a t-test (Fig 2). In particular, a region on chromosome 10 (81.7~82.2 Mb) was enriched for several differentiated SNPs. In total, we found 56 SNPs having estimated allele frequency differences above 0.25, with four of them above 0.4 (Fig 2).

## Validation of SNPs with large allele frequency differences

We selected the 14 SNPs with the highest estimated allele frequency differences in the 9 detected candidate regions for individual genotyping validation, with an emphasis on the chr10:81.7~82.2 Mb region (with 6 SNPs); the individually genotyped SNPs are listed in Table 1 and also indicated in Fig 2. Each of the 14 SNPs was typed in the full set of individuals, not just those used in constructing the pools (see Materials and Methods). Observed allele frequencies were calculated by genotype counting and we performed a Fisher's exact test for significant differences in allele frequencies, correcting for multiple testing. We used a very conservative cutoff of 0.5 x 10^–8^, assuming 1 million random markers and a single test level of 0.05. With this threshold, five out of the six SNPs from the region on chr10:81.7~82.2 Mb stood out as highly significant in the HL-LL as well as the Aymara-Guarani comparisons (Table 1). It is worth noting that Aymara and Quechua had very similar allele frequencies at these 14 SNPs, supporting the merging of these two groups into a single highlander group (Table 1) for further analysis.

**Table 1:** List of 14 SNPs with the highest allele frequency differences (estimated from pooling data) between highlanders (Aymara and Quechua) and lowlanders (Guarani).

#SNP	dbSNP ID	Chr.	Physical Position	Cytoband	Pooling |Δf HL|	IG |Δf HL|	IG P HL	IG P AG	IG P QG	IG P AQ
1	rs1489992	3	68805024	p14.1	0.41	0.45	3.9E-07	6.0E-06	7.3E-06	4.1E-01
2	rs11951359	5	58275600	q11.2	0.41	0.32	8.0E-05	8.2E-05	4.7E-03	7.0E-01
3	rs2544165	7	133920443	q33	0.34	0.39	3.0E-06	2.4E-05	1.3E-04	4.7E-01
4	rs4556111	8	113382838	q23.3	0.32	0.42	1.4E-06	5.2E-06	2.0E-04	8.3E-01
5	rs6476804	9	4083665	p24.2	0.39	0.49	7.4E-08	1.2E-07	6.6E-05	8.3E-01
6	rs2181204	10	81704512	q22.3	0.32	0.52	**2.6E-11**	**4.6E-10**	5.2E-07	1.0E+00
7	rs726289	10	81706951	q22.3	0.31	0.52	**2.4E-11**	**7.6E-10**	2.6E-07	7.6E-01
8	rs10788338	10	81733022	q22.3	0.36	0.35	3.5E-05	3.5E-04	1.2E-04	2.3E-01
9	rs12256429	10	81938632	q22.3	0.40	0.50	**1.5E-10**	**6.3E-09**	6.4E-07	5.6E-01
10	rs12779955	10	81940864	q22.3	0.43	0.51	**5.7E-11**	**2.0E-09**	2.6E-07	5.6E-01
11	rs8735	10	82192713	q23.1	0.42	0.65	**1.9E-14**	**2.3E-13**	**1.6E-08**	1.0E+00
12	rs3740393	10	104636655	q24.32	0.34	0.41	7.2E-07	1.2E-06	7.2E-04	6.5E-01
13	rs6497650	16	23132393	p12.1	0.38	0.39	7.3E-07	8.2E-06	8.2E-05	5.6E-01
14	rs10871349	16	78361149	q23.1	0.30	0.17	5.4E-02	9.8E-02	1.0E-01	8.4E-01

Chr. stands for Chromosome; Physical Position is from NCBI Build 37; Pooling |Δf HL| represents estimated absolute allele frequency difference from pooling experiments; IG |Δf HL| represents individually genotyped (IG) absolute allele frequency difference; P stands for P value; HL, AG, QG, AQ represent comparisons of highlander-lowlander, Aymara-Guarani, Quechua-Guarani, and Aymara-Quechua respectively. P values in bold are significant for Fisher's exact test with respect to the threshold of correction for multiple testing (i.e., 0.5E-08).

## Signals of population differentiation and positive selection

As the region on chromosome 10 (spanning approximately 500 kb from 81.7 to 82.2 Mb) contained several SNPs exhibiting significant allele-frequency differences between high- and lowland populations (Table 1), we investigated this signal in more detail. We performed targeted resequencing of a 1.5 Mb segment (from 81.1 to 82.6 Mb) that encompassed this region (see Materials and Methods) in 20 Aymara, 18 Quechua and 20 Guarani. The average coverage was 9X, and 1,983 SNPs were identified.

When population differentiation between the lowlander Guarani and the two highlander groups was examined, both the AYM-GUA and QUE-GUA comparisons revealed extreme *F*_ST_ values [45] that were generally above 0.5, reaching peak values of ~0.7 in the chr10:82.0~82.3 Mb region (S2A Fig). By contrast, the differentiation between the two highlander groups was much lower, with the *F*_ST_ values generally below 0.1 in this region (S2A Fig). This suggests that Aymara and Quechua may have shared very similar demographic/selection events, and that the extreme differentiation between the highlanders and Guarani in this region is unlikely to be accounted for solely by neutral genetic drift.

To formally test the latter hypothesis, we computed neutral simulations assuming several different demographic scenarios. Previous studies showed that the peopling of the Americas occurred more than 15,000 years ago through Beringia [46–48], with the initial colonization of the Andes around 11,000 years ago [6]. An admixture graph of Native American populations suggested that Guarani are descendants of Amazonia ancestry, which separated from high-altitude populations before the latter occupied the Andes [49]. To simplify the model, we set the divergence time between high and low-altitude populations at 10,000 years ago, and the divergence of the two high-altitude populations at 5,000 years ago. Population size estimates were obtained by using the demographic trajectory of Mexicans based on 1000 Genomes data [50] (red line in S3 Fig) as a surrogate for the common ancestral history of Quechua, Aymara and Guarani (see Materials and Methods). Given the much smaller recent population sizes of these three groups compared to Mexicans, we set constant population sizes of 9,000 after divergence for Quechua, Aymara and Guarani, instead of the sharp growth in the Mexicans. This scenario is referred to as the standard demographic model (green line in S3 Fig). We also simulated a bottleneck model with half the recent population sizes (blue line in S3 Fig), and a constant population size model (purple line in S3 Fig).

None of these neutral models could account for the strong divergence observed between Guarani and the two highlander groups in the chromosome 10 candidate region. The significant divergence signals strongly support the occurrence of positive selection, either in HL, LL, or in both groups. Table 2 lists the top 5% thresholds of *F*_ST_ values in simulations under all models (see Materials and Methods). Aymara and Quechua revealed an obvious lack of derived alleles with intermediate frequencies, particularly around the 82.0~82.3 Mb region of chromosome 10. Guarani did not seem to show any specific patterns (S2B Fig).

**Table 2:** The top 5% threshold of FST values for all comparisons in simulations under different demographic models.

Models	standard	Extreme bottleneck	Constant Ne
AYM-GUA	0.148	0.251	0.111
QUE-GUA	0.155	0.248	0.118
AYM-QUE	0.078	0.109	0.085
HL-LL	0.139	0.24	0.102

We also calculated Tajima’s D [51] and Fay and Wu’s H [52] for the resequenced data (see Materials and Methods). Interestingly, in both Aymara and Quechua, when compared to the standard demographic model, Tajima’s D values are marginally significant (P values = 0.0568 and 0.0565, S2C Fig) around chr10:82.0~82.3 Mb, and Fay and Wu’s H values are significant in both highlander groups in the same region (P values = 0.014 and 0.0212, S2D Fig). Guarani exhibits sporadic signs of selection around region 81.2 Mb and 82.6 Mb, but the signals are not consistent between Tajima’s D and Fay and Wu’s H (S2C and S2D Fig).

**Fig 3:**
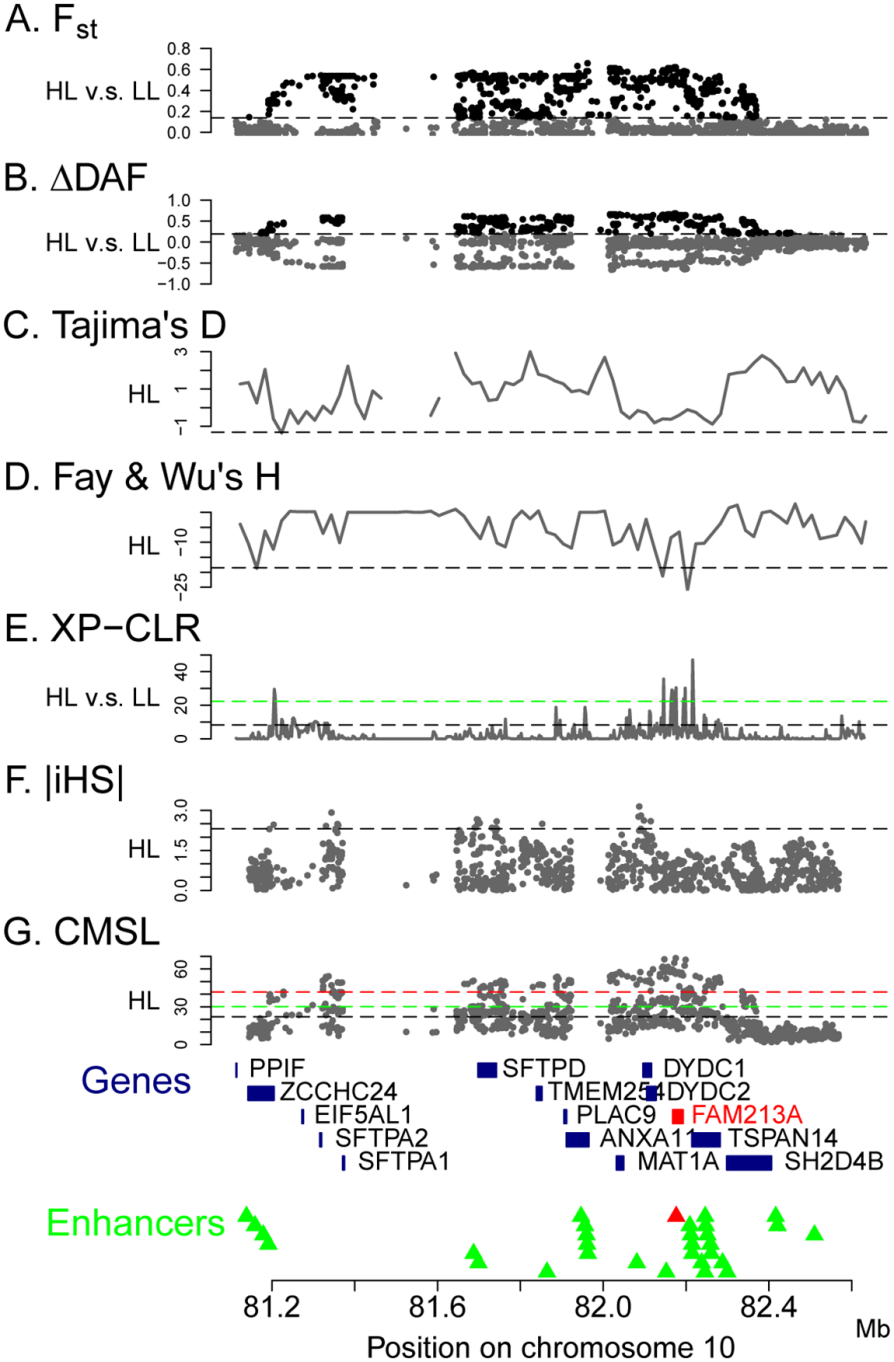
Signature of positive selection in highland populations, as revealed by various tests. (A) *F*_ST_ between HL and LL. (B) Derived allele frequencies of HL. (C) Tajima’s D of HL. (D) Fay & Wu’s H of HL. (E) XP-CLR of HL against LL. (F) Absolute iHS score of HL. (G) CMSL score of HL. Black, green, and red dashed lines are 5%, 1%, and 0.1% thresholds respectively of each test in standard simulations. *FAM213A* gene and its enhancer, covered by the peak of the CMSL scores, are labeled in red.

Since the genic region chr10:82.0~82.3 Mb in Aymara and Quechua hosts the strongest and most consistent signals of selection, and the genetic profiles in this region are highly similar between Aymara and Quechua as shown in previous studies [33,43,44], we carried out in depth analyses of positive selection on the merged HL data. First, a composite of multiple signals like (CMSL) test was constructed based on six different selection tests: *F*_ST_, ΔDAF [53], Tajima’s D, Fay & Wu’s H, XP-CLR [54] and iHS [55] (see Materials and Methods). As can be seen in Fig 3, individual tests in general showed consistent signals of positive selection in this region. The patterns of *F*_ST_, ΔDAF, Tajima’s D, and Fay & Wu’s H are highly similar in Aymara and Quechua (Fig 3A–3D and S2 Fig); however in Guarani there is no consistent pattern (S4 Fig). XP-CLR and iHS both exhibit the highest signals within the 82.0~82.3 Mb interval although the iHS peak locates upstream from that of XP-CLR (Fig 3E and 3F). The maximum XP-CLR value is 46.99 (P value = 9.01 x 10^–4^) and maximum |iHS| is 3.144 (P value = 0.00732, Fig 3E and 3F). When all these signals are combined together to derive a summary CMSL score (see Materials and Methods), the empirical CMSL scores are highly significant compared to the neutral CMSL distribution obtained from the simulations under the standard demographic (Fig 3G), extreme bottleneck (S5A Fig), and constant size models (S5B Fig). Signals in both individual tests and the CMSL test are consistently located within the 82.0~82.3 Mb interval, which provides strong evidence of positive selection in HL. The CMSL scores narrow the signal to a ~57 kb region that contains one protein coding gene, *FAM213A*, and an enhancer that significantly influence the expression of this gene [56]. The peak region of CMSL is similar under all models (Fig 3G and S5 Fig), and the enhancer (chr10:82176099–82176325) is close to the highest CMSL signal (chr10:82174949).

**Fig 4:**
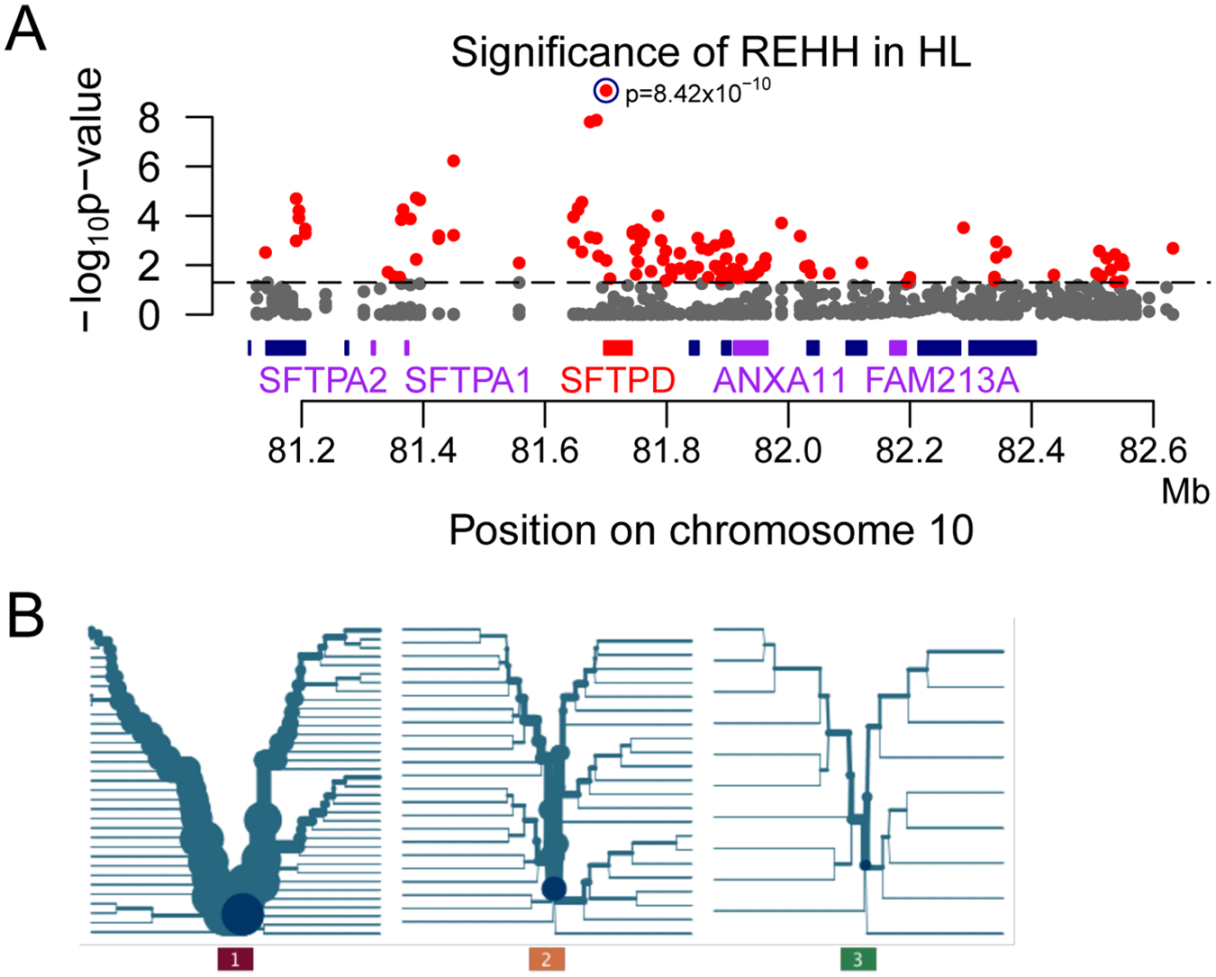
REHH results of HL and the most significant core haplotype. (A) P value of each haplotype based on standard simulations. (B) The most significant core haplotype, located in the *SFTPD* gene. Haplotype 1 reaches a frequency of 52.6% and is inferred to be the haplotype with the most significant signal of selection.

Another test commonly used to identify candidate regions of positive selection is the relative extended haplotype homozygosity (REHH) test, which is based on the principle of long extended haplotypes [57]. We scanned the entire candidate region and found widespread REHH signals (Fig 4A). The strongest signal occurred between position chr10:81699238 and chr10:81701722, within the *SFTPD* gene, where a major core haplotype with a frequency of 52.6% (haplotype 1 in Fig 4B) decays much slower than the other two haplotypes (haplotype 2 and 3 in Fig 4B). The P value for the observed excessive EHH of haplotype 1 is 8.4 x 10^–10^, indicating a strong signal of positive selection.

## Discussion

Nowadays, it is estimated that worldwide some 140 million [58] people reside permanently at an altitude of 2,500 meters or more above sea level, and that countless others sojourn to high plateaus and mountainous regions for leisure or professional activities. The physiology of humans living at high altitude has been the subject of over a century of research, especially in Tibetan, Ethiopian and Andean populations which have acquired long-term physiological, anatomical, and biochemical responses to high-altitude environmental stress when compared to lowland inhabitants. Recent advances in genomic technologies are providing opportunities to explore the genetic basis of their adaptive traits, particularly in the regulatory systems of respiration and circulation.

In the present study, we focused on three populations from Bolivia, namely two groups of native inhabitants of the Andes: Aymara and Quechua, and Guarani from the Gran Chaco lowlands as a neighboring control group. Special care was taken to obtain samples from members of long-term high-altitude residence families, avoiding the collection of recent immigrants. Moreover, we collected samples only from healthy individuals, especially with no known medical record or indication of CMS

We performed a genome-wide scan of over 900,000 SNPs using microarray technology on pooled DNA samples (see Results), thus examining the genetic profile of each group in the search for large differences in allele frequencies among them. After validation of the individual genotypes for the variants exhibiting the largest allele frequency differences (on average more than 38%), we applied multiple-test corrections and detected a region in chromosome 10 harboring several SNPs that achieved statistical significance. Genotyping of pooled samples decreases the power to detect weaker signals of population differences; however the fact that we do detect a strong signal of population differentiation that is likely to be due to selection further substantiate the utility of pooled data in genome scan studies [38–41].

The region on chromosome 10 is a novel candidate region for high-altitude adaptation, which has not been detected in previous studies. We further verified that the signal of high differentiation between HL and LL groups for the chromosome 10 region was unlikely to arise by demographic events alone after carrying out simulations under various demographic models. In order to investigate in more detail the potential signal of selection in chromosome 10, we performed targeted resequencing of ~1.5 Mb surrounding the region of interest. Table 3 lists all protein coding genes in the candidate region and all non-synonymous SNPs observed in the resequencing data; regulatory elements could also be the target of positive selection, and several enhancers are included in the candidate region (Fig 3G and S1 Table).

**Table 3:** Protein coding genes in the candidate region. DAF stands for derived allele frequency.

Gene	Description	Amino acid changes	DAF_HL	DAF_LL	*F*_ST_
PPIF	peptidylprolyl isomerase F				
ZCCHC24	zinc finger, CCHC domain containing 24	chr10:81192404, Arg/stop	0.026	0.000	0.001
EIF5AL1	eukaryotic translation initiation factor 5A-like 1				
SFTPA2	surfactant protein A2				
SFTPA1	surfactant protein A1				
SFTPD	surfactant protein D	rs3088308, Ser/Thr, D^a^	0.118	0.700	0.537***
		rs2243639, Thr/Ala	0.526	0.200	0.181*
		rs721917, Met/Thr	0.684	0.280	0.271**
TMEM254	transmembrane protein 254	rs1932574, Cys/Phe	0.316	0.080	0.133
PLAC9	placenta-specific 9				
ANXA11	annexin A11	rs1049550, Arg/Cys, D	0.671	0.150	0.408***
MAT1A	methionine adenosyltransferase I, alpha				
DYDC1	DPY30 domain containing 1				
DYDC2	DPY30 domain containing 2				
FAM213A	family with sequence similarity 213, member A				
TSPAN14	tetraspanin 14				
SH2D4B	SH2 domain containing 4B	rs7075840, His/Arg	0.789	0.550	0.112
		rs17107368, Asp/Glu	0.026	0.050	-0.011

^a^. predicted to be “damaging” by Polyphen.

* *P* < 0.05

** *P* < 0.01

*** *P* < 0.001

The strongest evidence of positive selection was in the region of 82.0~82.3 Mb in chromosome 10, where several genes (*ANXA11*, *MAT1A*, *DYDC1*, *DYDC2*, *FAM213A*, *TSPAN14* and *SH2D4B*) are included in or near the borders of this region (Fig 3G). One non-synonymous SNP (rs1049550) was detected in the *ANXA11* gene, is predicted to be ‘damaging’ by Polyphen [59], and showed a significant differentiation between highlanders and lowlanders. Strong associations have been repeatedly found between genetic polymorphisms of *ANXA11* and sarcoidosis, a systemic immune disorder characterized by destructive, noncaseating epithelioid granulomatous lesions (i.e., nodules caused by inflammation that do not lead to cell death) [60–62]. It is most often located in the lung or associated lymph nodes. The sarcoidosis-associated SNPs are listed in Table 4. In addition, a genome wide association study of chronic obstructive pulmonary disease identified one SNP in an intron of *ANXA11* [63]. The risk allele is rs6585424-G (Table 4, P value = 1 x 10^-10^).

**Table 4:** Risk alleles identified by association studies (GWAS Catalog [64]).

Genes	PubMed	Trait	Risk allele	P value	context
PPIF	20881960	height	rs2145998-A	4 x 10^–13^	intergenic
			rs7916441-?	6 x 10^-10^(Conditioned on rs2145998)	intron
SFTPD	23144326	Chronic obstructive pulmonary disease-related biomarkers	rs3923564-G	2 x 10^–27^	intron
			rs7078012-T	5 x 10^–9^	intron
ANXA11	23144326	Chronic obstructive pulmonary disease-related biomarkers	rs6585424-G	1 x 10^–10^	intron
	22936702	Sarcoidosis	rs1953600-?	1 x 10^–6^	intergenic
	19165924	Sarcoidosis	rs2789679	3 x 10^–13^	intergenic
			rs7091565	1 x 10^–5^	intergenic
TSPAN14	23128233	Inflammatory bowel disease	rs6586030-G	9 x 10^–16^	intron
SH2D4B	22864933	Capecitabine sensitivity	rs6586111-?	7 x 10^–6^	intron

The FAM213A gene was localized by the CMSL test under all models of population history (Fig 3G and S5 Fig). Also known as PAMM: peroxiredoxin (PPX)-like 2 activated in M-CSF-stimulated monocytes [65], it has been shown that the expression of FAM213A can protect cells from oxidative stress and modulate osteoclast differentiation through inhibition of NF-κB and c-Jun activation, which may affect bone resorption and help to maintain bone mass [65]. Oxidative stress is one of the most detrimental effects of hypobaric hypoxia, which is caused by increased reactive oxygen species (ROS), reactive oxygen and nitrogen species (RONS), decreased antioxidants and reduction in pulmonary nitric oxide (NO) bioavailability (reviewed in [66]). Antioxidant supplementation has been shown to have beneficial effects and reduced the oxidative stress of some individuals [67]. The expression levels of antioxidants were upregulated in hypoxia tolerant rats [68], and also in sojourners after a high-altitude stay, even if not sufficient to ameliorate oxidative stress completely [69]. These studies suggest that antioxidants are quite important in protecting against oxidative stress, and adaptive effects on the antioxidant system could be influenced by genetic factors, which differ between highlanders and sojourners. Moreover, as a consequence of preventing oxidative damage, the expression of FAM213A could abolish osteoclast formation, resulting in the maintenance of bone mass. It is unclear if this function of FAM213A would be beneficial for high-altitude adaptation; however, studies have shown accelerated growth in lung volume and chest dimensions in highlanders vs. lowlanders [70–72], which might be a developmental compensatory response to high-altitude hypoxia [73].

In addition to the FAM213A gene itself, the target region of positive selection includes an enhancer of FAM213A. Two SNPs are located in this enhancer; one (rs77999529) exhibits a low minor allele frequency in various human populations, while the second (rs150230265) exhibits significant allele frequency differences between HL and LL (FST = 0.229, P value = 0.014, S1 Table). The global distribution of the allele frequencies of rs150230265 is shown in S6 Fig, which suggests that the derived G allele is restricted to Native American populations. Moreover, the derived allele is at highest frequency (0.382) in HL, and hence could be considered a candidate mutation. The fact that the rs150230265 SNP does exist at low frequency in low-altitude Native American populations (but nowhere else) makes selection on standing variation a possibility, which would further reduce the signal of selection in tests for selective sweeps. Our results suggest that elevated expression of FAM213A by positive selection on the enhancer could help protect against oxidative damage in a hypoxia environment. The mutation and the enhancer could thus be novel candidates for further experimental studies and therapeutic targets.

Although FAM213A was detected as a candidate gene in the CMSL analysis, it was not identified by the REHH analysis. Instead, a different candidate gene in the resequenced region, the SFTPD gene, was identified by this analysis. These different results are not surprising, given that different methods have different power to detect selection, especially in the case of partial selective sweeps and/or selection on standing variation [74]. SFTPD encodes lung surfactant protein D (SP-D), which contributes to the lung’s defense against inhaled microorganisms and may participate in the extracellular reorganization or turnover of pulmonary surfactant. Pulmonary surfactant in turn lowers the surface tension at the air-liquid interface in the alveoli of the mammalian lung and is essential for normal respiration. Given the low oxygen levels at high altitude, altering the surfactant surface tension could be beneficial. A genome-wide association study of chronic obstructive pulmonary disease identified two risk alleles in an intron of SFTPD: the G allele of rs3923564 (P value = 2 x 10^–27^) and the T allele of rs7078012 (P value = 5 x 10^–9^) [63]. Several non-synonymous SNPs in SFTPD were detected in our resequencing data (Table 3). The rs3088308 SNP involves a serine to threonine substitution, was predicted to be ‘damaging’ by Polyphen, and exhibits significant differentiation between HL and LL (FST = 0.537, P value = 5.46 x 10^–5^). However, the derived allele frequency is higher in LL than in HL. Another SNP (rs721917) involves a methionine to threonine substitution and exhibits significant differentiation between HL and LL (FST = 0.271, P value = 7.6 x 10^–3^) with a higher frequency of the methionine-encoding allele in HL. This mutation has been investigated intensively and influences oligomerization, function, and the concentration of SP-D in serum [75]. The Thr/Thr genotype had significantly lower SP-D serum levels, and is associated with increased disease-susceptibility [76–78]. The Met allele was associated with defense to respiratory syncytial virus [76]. The third non-synonymous SNP is rs2243639 (Thr/Ala), which also showed significant differentiation between HL and LL (FST = 0.181, P value = 0.028).

In addition to SFTPD, there are two other genes coding for surfactant pulmonary-associated proteins (SFTPA1 and SFTPA2) which are within the genomic region resequenced, but outside the region showing the highest signals in the CMSL and REHH tests. Mutations in SFTPA1 and SFTPA2 are associated with idiopathic pulmonary fibrosis [79], and (along with SFTPD) play an essential role in surfactant homeostasis and in the defense against respiratory pathogens [80,81]. Given that these surfactant proteins play a role in both lung function and disease resistance, it is unclear which of these (or perhaps both) might be the driving force behind the signals of selection that we detect in the HL populations.

The novel candidate genes for high-altitude adaption identified here are in accordance with previous evidence that the functional adaptations of Andean, Tibetan, and Ethiopian natives to high altitude differ [11]. Andeans exhibit lower levels of resting ventilation, a more ‘blunted’ HVR, higher levels of pulmonary hypertension and an increased frequency of CMS. In Tibetans, the exhaled NO is elevated compare to Andean and lowlanders [82], which was associated with higher blood flow through the lung [83]. Similar hemoglobin phenotypes among Tibetan and Ethiopian highlanders associate with different genetic loci, and the variants at those loci are present in most populations regardless of altitude [84]. Overall, populations in different continents have adapted to high altitude through different adaptation processes as a result of convergent evolution [85,86].

A recent study showed that altitude adaptation in Tibetans may have arisen via introgression of Denisovan-like DNA [87]. Thus, modern humans could obtain genetic adaptations to local environments through admixture with other hominin species [88–90]. Native American populations migrated from Siberia, where admixture might have happened between ancestors of modern Asians and archaic humans (including Neanderthals and Denisovans) [91–93]. We therefore checked our sequence data and found no haplotype specifically shared with Denisovans in the region surrounding both *FAM213A* and *SFTPD* genes (S7 Fig). These results further support different routes to functional adaptation in Tibetan and Andean high-altitude natives [11].

In summary, we identified a novel candidate region for high-altitude adaptation in Andeans, with several genes and/or enhancers potentially under positive selection. In particular, multiple tests localized the signal to *FAM213A* and a related enhancer encoding an antioxidant to reduce oxidative stress, which might be beneficial for adaptation to high altitude in the Andes. However, further functional studies are needed to elucidate the role of this gene (as well as the other candidates) in high-altitude adaptation.

## Materials and Methods

### Sample collection and DNA extraction

We collected in total 99 saliva samples from South American indigenous individuals from Bolivia. The participants were informed about our study objectives and provided written consent for the anonymous use of the biological material for academic research. This research was approved by the Ethics Committee of the University of Leipzig Medical Faculty. All sampled individuals were unrelated and of self-identified ancestry as either Aymara, Quechua or Guarani. They were members of long-term residence families from the places where samples were gathered; sample collection from recent immigrants was avoided. Furthermore, special care was taken to obtain samples only from healthy individuals, with no known medical record or indication of CMS or other altitude-related illness. The Aymara individuals were sampled in El Alto (N = 24, situated at 4,100 m altitude above sea level), Tiwanaku (N = 24, 3,885 m), and La Paz (N = 7, 3,600 m). The Quechua individuals were sampled in Oruro-Soracachi (N = 21, 3,750 m), and the Guarani individuals were sampled in Santa Cruz-Gran Chaco (N = 23, 416 m). Genomic DNA was extracted from the saliva samples following the protocol published elsewhere [94], and the fraction of endogenous (i.e., human) DNA present in the extracts was quantitated as described previously [94].

### Y chromosome haplogroups

A total of 56 males from our Bolivian collection of samples were genotyped for 24 SNPs (12f2, M106, M124, M145, M168, M170, M172, M174, M175, M20, M201, M207, M213, M214, M269, M45, M52, M69, M9, M91, M96, MEH2, SRY10831, and Tat) defining the major branches of the Y chromosome tree [95]. The 24 loci were typed and used for haplogroup assignment as described in [96].

### DNA pooling and microarray genotyping

To search for candidate genomic regions of high differentiation between high and low-altitude Bolivian populations, we genotyped pooled samples on microarrays; this approach has been used successfully in other studies [38–41]. A total of nine equimolar DNA mixtures were constructed, consisting of one pool of 18 Aymara, one pool of 17 Quechua, and one pool of 18 Guarani samples; each pool was prepared independently in triplicate with the same individuals, thus resulting in three technical replicates for each pool. We selected the individual genomic extracts containing the highest fractions of endogenous DNA, with all selected extracts containing ≥ 30% human DNA [97]. Each individual sample contributed 100 ng human DNA to the mixture. Pooled DNA solutions were diluted to a working concentration of 50 ng/μl with ddH2O. Affymetrix Reference Genomic DNA 103 was used as a positive control for the microarray experiments. Genotyping was performed using the Affymetrix Genome-Wide Human SNP Array 6.0 according to the manufacturer's protocol. Each of the nine DNA pools and the positive control sample were assayed on a separate microarray. Each array was scanned using the Affymetrix GeneChip Scanner 3000 with the High-Resolution Scanning Upgrade. The cell intensity files were analyzed using the Affymetrix Genotyping Console (GTC v2.1), and the concordance of called genotypes (excluding missing data) between replicates and between the positive control and its consensus genotypes provided by Affymetrix was analyzed using GTC v2.1. The concordance for the pooled Aymara genotypes was 97.5% (on average for the pairwise comparison among the three replicates), for the Quechua was 96.7%, and for the Guarani was 97.9%. The concordance of the positive control compared to the consensus genotypes provided by Affymetrix was 99.7%.

### Allele frequencies from DNA pools and highly differentiated genic regions

The allele frequency per called SNP and population was estimated from the raw probe intensity data of each microarray as previously described [38–40]; the allele frequency data are available from the authors upon request. Briefly, we computed the Relative Allele Signal (RAS) score as an estimate of the allele frequency. In order to have consistent calculations, we only considered the first three probe sets for each SNP locus and removed SNPs whose standard deviation of RAS across technical replicates and/or probe sets in any group of individuals was greater than 0.1. Then, the allele frequency for each group of individuals was estimated by averaging across the technical replicates and the probe sets:$\bar{p}=\frac{\sum_{j}\sum_{k}RAS_{j,k}}{n_{j}n_{k}}$, where *j* is the technical replicate and *k* is the probe set. The allele frequency difference was taken as $\left|{\bar{p}}_{1}-{\bar{p}}_{2}\right|$, where *p*_*1*_ and *p*_*2*_ are the allele frequencies in two different groups. We calculated the allele frequency differences between groups in a pairwise fashion, and we also compared the Guarani against Aymara and Quechua individuals combined together into a single highland group.

We applied a t-test to formally evaluate the statistical significance of the calculated allele frequency differences. Therefore, $T=\frac{{\left({\tilde{p}}_{\text{1}}-{\tilde{p}}_{\text{2}}\right)}^{\text{2}}}{\text{var}({\tilde{p}}_{\text{1}}-{\tilde{p}}_{\text{2}})}$, where *T* should follow a $\chi_{\text{1}}^{\text{2}}$ distribution. The overall variance: $\text{var}({\tilde{p}}_{\text{1}}-{\tilde{p}}_{\text{2}})$ consists of two parts: *V*_s_+*V*_p_, where *V*_s_ represents the sampling variance, and *V*_p_ represents the component from the pooling process. *V*_s_ is given by $\frac{{\tilde{p}}_{\text{1}}(\text{1}-{\tilde{p}}_{\text{1}})}{{\text{2n}}_{\text{1}}}+\frac{{\tilde{p}}_{\text{2}}(\text{1}-{\tilde{p}}_{\text{2}})}{{\text{2n}}_{\text{2}}}$ and *V*_p_ is given by $\frac{\text{var}({\tilde{p}}_{\text{1}})}{{\text{2n}}_{\text{1}}}+\frac{\text{var}({\tilde{p}}_{\text{2}})}{{\text{2n}}_{\text{2}}}$, where *n* is the sample size.

We applied a multi-locus approach to search for highly differentiated genic regions. SNPs were ranked according to either the allele frequency difference or P value significance. The top 0.1% SNPs were connected if they were within 100 kb distance, and a differentiated region was called if there were more than 10 top SNPs connected.

### Validation of estimated allele frequencies

Confirmation of the allele frequencies estimated from the RAS scores for the 14 SNPs with the largest HL-LL allele frequency differences was performed using the ABI PRISM SNaPshot Multiplex System (Applied Biosystems by Life Technologies) according to the manufacturer's protocol. Primers for the single PCRs and for the subsequent extension reactions were designed using the UCSC In-Silico PCR tool (http://genome.csdb.cn/cgi-bin/hgPcr/). Primer interactions within the multiplex were evaluated and minimized using the NetPrimer online software (http://www.premierbiosoft.com/). Briefly, single PCRs amplified the target region surrounding the SNP of interest for each individual contained in the full collection set. The amplicons were then assembled into four separate multiplexes and analyzed on an ABI 3130xl Genetic Analyzer. The SNP calling was performed using the ABI GeneMapper ID v3.2 software. As a positive control for the SNaPshot experiments, the sample HapMap #NA06985 CEPH/UTAH Pedigree 1341 was assayed along with the Bolivian samples. The called genotypes for the control were compared with the consensus genotypes for the same 14 SNP loci obtained from the HapMap website; the concordance was 100%. Additionally, one Bolivian sample was assayed in single primer extension reactions for each of the 14 SNPs, and the called genotypes were compared to the genotypes obtained from the multiplex approach; the concordance was 100%. For the 14 SNPs re-genotyped individually, we performed a Fisher’s exact test to validate the results obtained from the DNA pooling approach. The Fisher’s exact test was performed using R (http://www.r-project.org/).

### Capture array and resequencing

We used Agilent custom 1M capture arrays in order to resequence the target region of interest. We designed overlapping microarray probes of 60 bases targeting over 1.5 Mb of the region of interest in chromosome 10 (chr10:81113000–82664000). Probes were tiled every 3 bases across the target region. Probes containing repetitive elements were discarded [98]. We used the human reference sequence NCBI Build 37 (hg19) to design the probes.

Illumina GAIIx libraries were prepared following Meyer and Kircher [99], with some differences noted below. All samples were sheared with the Bioruptor UCD-200 (Diagenode) down to a range of approximately 200–800 bps. The adapter fill-in step was performed using Dynabeads MyOne Streptavidin C1 (Invitrogen). The beads were prepared and libraries immobilized by aliquoting 25μl bead suspensions for each sample, washing twice with 2X-BWT buffer and eluting in 25μl 2X-BWT buffer. A magnetic plate was used for all washing steps. The adapted sample libraries were added to the bead suspension, pipette-mixed, and incubated for 15 minutes at room temperature. The supernatant was then discarded while the plate was on a magnet and the beads were washed twice with 100μl 1X-BWT buffer. The fill-in step was performed by adding the master mix used in Meyer and Kircher [99] after removing the buffer, and no subsequent SPRI purification was necessary.

Individual-specific indexes were used to multiplex the libraries prior to hybridization enrichment. These were attached by performing a PCR amplification using the Phusion Mastermix (New England Biolabs, NEB). After indexing, samples were pooled in equimolar ratios and hybridized. After hybridization, quantitative PCRs were performed on the sample pools with the DyNAmo qPCR kit (NEB). Based on the resulting qPCR amplification plots, the sample pools were amplified using the Phusion Mastermix so that they did not reach plateau. Each sample pool was sequenced on a single lane of an Illumina GAIIx run by single-end sequencing using 36 cycles.

### Resequence data processing

The raw sequencing reads were aligned to the human reference genome sequence GRCh37 by BWA v0.70 [100] with default parameters. The alignments were transferred to indexed binary alignment map (BAM) files by SAMtools [101] and duplicates removed with the Picard tool v.1.66.

Genotypes were called by the GATK UnifiedGenotyper v1.4 [102] with the following parameters: the minimal base quality score setting was 20, the minimal mapping quality score setting was 30, and the confidential Phred-scale threshold for genotyping calling setting was 50; the default settings were used for all other parameters. Furthermore, the GATK VariantRecalibrator tool was used to score variant calls by a machine-learning algorithm and to identify a set of high-quality SNPs using the Variant Quality Score Recalibration (VQSR) procedure. The insertions and deletions (indels) were filtered by GATK, resulting in 1,983 SNPs (with average coverage 9X) for the following analyses.

### Population Genetic Analyses and Selection Tests

To analyze the population differences and detect signals of natural selection in either high or low-altitude populations, we employed several methods with both empirical polymorphism data and simulated data. These methods were based on population differentiation, the allele frequency spectrum, properties of haplotypes, and composite signals:

#### FST test

*F*_ST_ is a measurement of population differentiation. We calculated it in pairwise manner by using the unbiased estimator of Weir and Cockerham [45].

#### ΔDAF test

We calculated ΔDAF [53] between a putative selected population and a non-selected population. ΔDAF scores range between -1 and 1. SNPs with positive scores indicate a higher derived allele frequency in the selected population. The ancestral allele states were as determined by the 1000 Genomes Project [50].

#### XP-CLR test

XP-CLR test is a likelihood method for detecting selective sweeps based on multilocus allele frequency differentiation between a putative selected population and a non-selected population [54]. We set 0.05 cM sliding window sizes and uniform grid points with a spacing of 2 kb. The maximum number of SNPs was set to 200 for each window.

#### Tajima’s D test

Tajima’s D [51] was performed with a sliding window of 20 kb and no overlap between adjacent windows. The calculation was performed by an in-house Perl script.

#### Fay&Wu’s H test

Fay & Wu’s H [52] was also calculated by an in-house Perl script with the same sliding window approach as in Tajima’s D test.

#### iHS test

This method is based on the length of the haplotype associated with ancestral vs. derived alleles; derived alleles subject to positive selection tend to have unusually long haplotypes, as such alleles have risen to high frequency too quickly for recombination and/or new mutations to break down the length of the associated haplotype. The iHS test partitions haplotypes into an ancestral group and a derived group according to the allele states of core SNPs; iHS is defined as the log ratio of the integrated EHH (extended haplotype homozygosity) for these two groups [55].

#### REHH test

The REHH is another test based on haplotype length and structure, and was calculated with the Sweep software [57]. We set the option ‘matching distance’ to be ‘marker H of about 0.04’.

#### CMSL test

Numerous methods have been developed to detect positive selection based on various patterns of genetic variation, and hundreds of candidate regions have been identified. But usually these regions are typically large and the causal variants remain unknown. A composite of multiple signals method narrows down the candidate regions and aids in identifying the causal variant [53]. Recently, another framework combing P values in large scale genomic data was used to detect selection [103]. This test is based on Fisher’s combination test [104]. The statistic is computed as $Z_{F}=-2\sum_{i=1}^{k}\log P_{i}$, where *k* is the number of SNPs in one region and *P*_*i*_ is the empirical P value of one test for the SNP *i*. In Luisi’s study, *F*_ST_, ΔDAF and iHS statistics are calculated, and regions with high Z_F_ scores indicate positive selection. Following this approach, we used a CMSL method by combining *F*_ST_, ΔDAF, Tajima’s D, Fay & Wu’s H, XP-CLR and the iHS test. The Z_F_ statistic is computed as above, where *k* is the number of tests and *P*_*i*_ is the P value of the SNP in test *i*. We obtained the P value from empirical distributions by simulations.

### Simulations

Simulations were used to calculate the P value of the scores in the empirical data. To account for the impact of demography on the detection of selection, we did simulations under a wide range of demographic scenarios inferred by pairwise sequentially Markovian coalescent (PSMC) model, which is a method to infer the history of population size change based on a single genome sequence [105]. In this study, we sequenced nearly 1.5 Mb, which is not enough for inferring a high resolution *N*_*e*_ trajectory. We therefore used the *N*_*e*_ estimated from the Mexican (MXL) population in the 1000 Genomes Project [50](red line in S3 Fig), with the modification of a constant *N*_*e*_ in recent history instead of a sharp expansion, as our standard demographic model. We set the divergence time between high and low-altitude populations at 10,000 years ago, and the divergence of the two high-altitude populations at 5,000 years ago. We also used two other models, one with a more intense bottleneck (*N*_*e*_ reduced by 50% during the most recent 10,000 years) and one with a constant *N*_*e*_ of 7,000 for the entire history.

We used a different formula for the time interval boundaries in PSMC:

$$t_{i}=0.1e^{{\left(\frac{i}{n}\right)}^{2}\log(1+10T_{\max})}-0.1,i=0,\ldots ,n$$

We set *n* to be 30, to reduce the complexity of the search space. The squared exponential growth of time intervals results in more intervals in the recent past and much fewer intervals in the ancient past, as recent *N*_*e*_ needs more information for accurate inference and is more important for our purposes. We simulated 2 Mb neutral segments with MSMS [106] with 100 replicates for each of the three demographic scenarios.

## Supporting Information

S1 Fig Y Chromosome haplogroup frequencies (%) in the analyzed individuals and populations. (TIF)

S2 Fig Population differentiation, derived allele frequencies and signature of selection in individual groups. (A) *F*_ST_ in all comparisons. (B) Derived allele frequencies. (C) Tajima’s D. (D) Fay & Wu’s H. Black dashed lines are the 5% threshold of corresponding tests from the standard simulations.
(TIF)

S3 Fig Demographic models used in simulations. The red line is the population size trajectory of MXL as estimated by PSMC. The green line is from the standard model modified from the MXL trajectory by assuming a constant population size in recent history. The blue line is the extreme bottleneck model modified from the standard model by reducing *N*_*e*_ by half beginning 10,000 years ago. The purple line is the constant model with a constant *N*_*e*_ of 7,000.
(TIF)

S4 Fig Signature of positive selection in the lowland population as revealed by multiple tests. (A) *F*_ST_ between HL and LL. (B) Derived allele frequencies of LL. (C) Tajima’s D of LL. (D) Fay & Wu’s H of LL. (E) XP-CLR of LL against HL. (F) Absolute iHS score of LL. (G) CMSL score of LL. Black dashed lines are the 5% threshold of each test in standard simulations, green lines are the 1% threshold, and red lines are the 0.1% threshold.
(TIF)

S5 Fig Signature of positive selection. CMSL in highland populations under (A) the extreme bottleneck model and (B) the constant population size model.
(TIF)

S6 Fig Global distribution of the allele frequencies of rs150230265. Data are from this study and 1000 Genomes.
(TIF)

S7 Fig Common haplotype frequency. Haplotype frequencies in modern humans (from this study and 1000 Genomes data) and Denisovan genome sequence in *FAM213A* and *SFTPD* (low quality Denisovan sites were filtered following [87]). Green is haplotype shared with Denisovan; blue is the first dominant haplotype in HL; red is the second dominant haplotype which contain the derived allele of rs150230265 in HL; purple is the first dominant haplotype in LL; gray are haplotypes with frequencies <10% in Bolivian populations. The radii are scaled by sample sizes. (A) Haplotypes are 10kb extended on both sides from rs150230265. (B) Haplotypes are 10kb extended on both sites from the three non-synonymous SNPs in *SFTPD*.
(TIF)

S1 Table Enhancers and associated genes in the candidate region. The ‘robust set’ enhancers and the ‘enhancer_tss_association’ were downloaded from the FANTOM5 project [56] (http://enhancer.binf.ku.dk/Pre-defined_tracks.html).
(XLSX)
