# Supplementary figures and images for: A Novel Candidate Region for Genetic Adaptation to High Altitude in Andean Populations
Source: PLoS One. 2015 May 11;10(5):e0125444. doi: 10.1371/journal.pone.0125444 (PMC4427407; doi:10.1371/journal.pone.0125444)

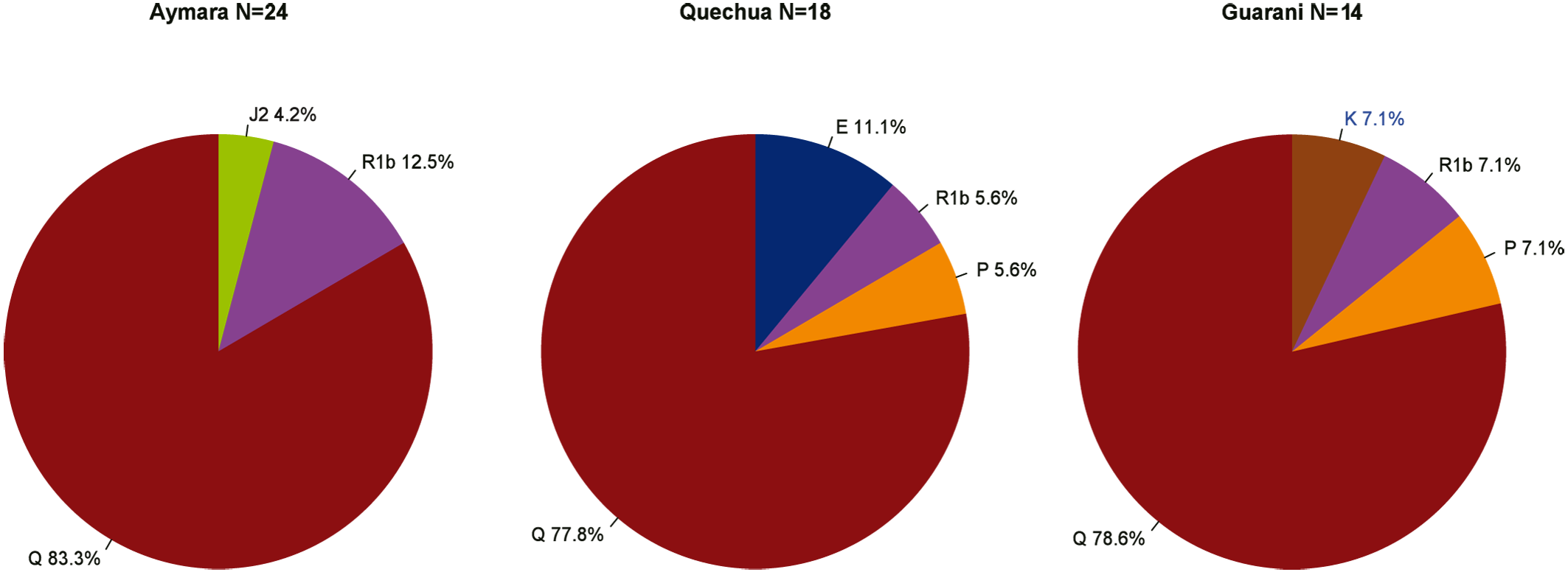

Supplement: S1 Fig — (TIF) [file pone.0125444.s001.tif]

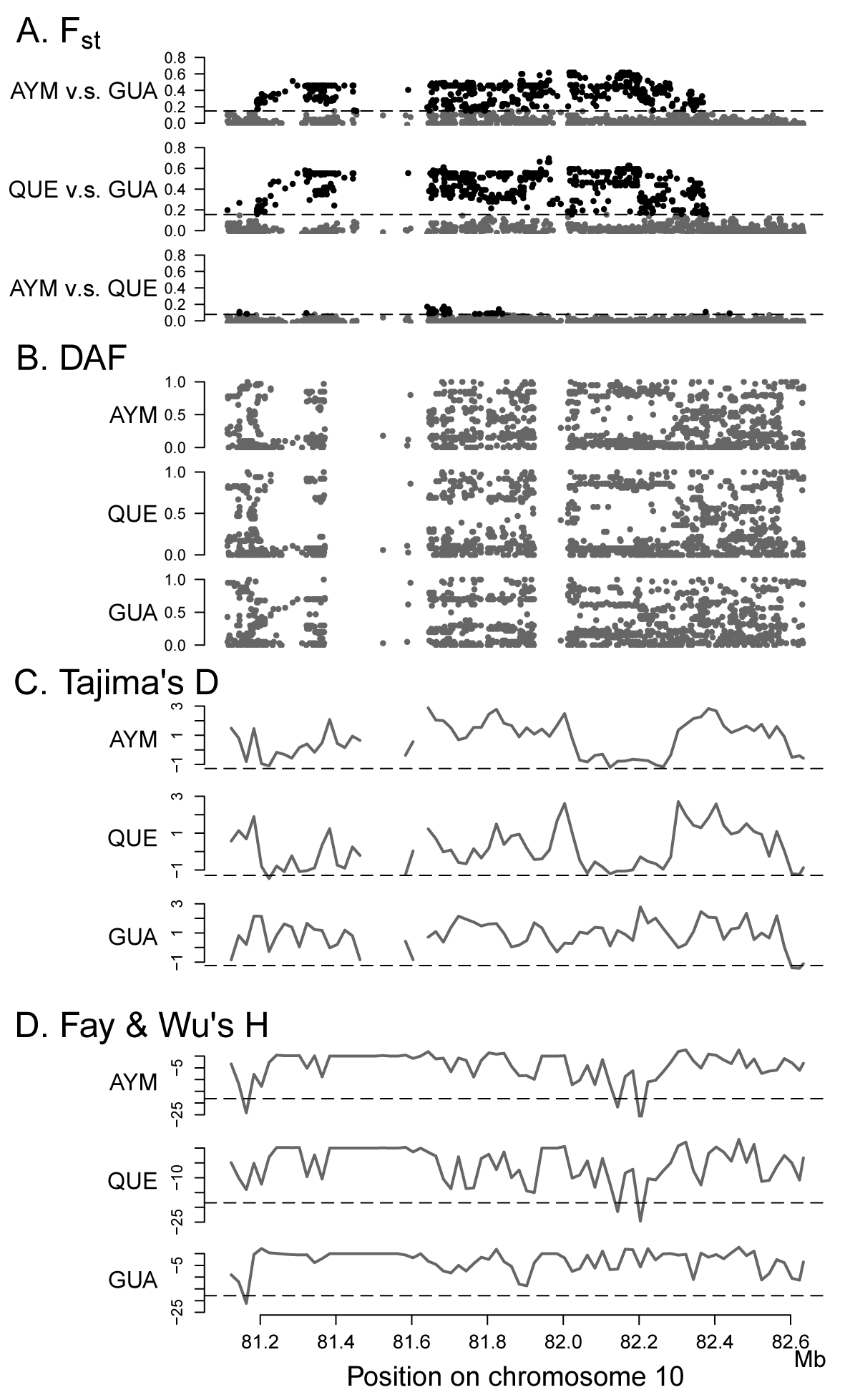

Supplement: S2 Fig — (A) F ST in all comparisons. (B) Derived allele frequencies. (C) Tajima’s D. (D) Fay & Wu’s H. Black dashed lines are the 5% threshold of corresponding tests from the standard simulations. (TIF) [file pone.0125444.s002.tif]

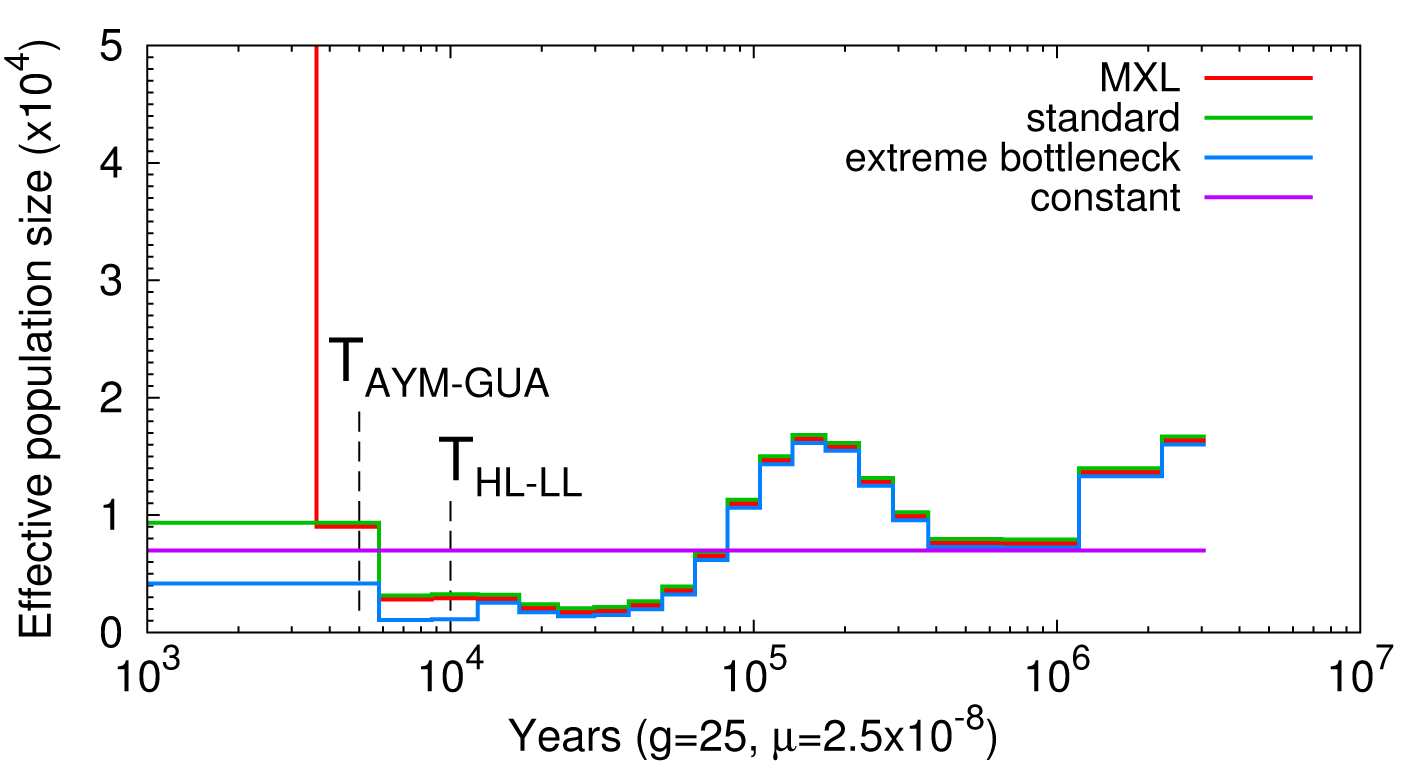

Supplement: S3 Fig — The red line is the population size trajectory of MXL as estimated by PSMC. The green line is from the standard model modified from the MXL trajectory by assuming a constant population size in recent history. The blue line is the extreme bottleneck model modified from the standard model by reducing N e by half beginning 10,000 years ago. The purple line is the constant model with a constant N e of 7,000. (TIF) [file pone.0125444.s003.tif]

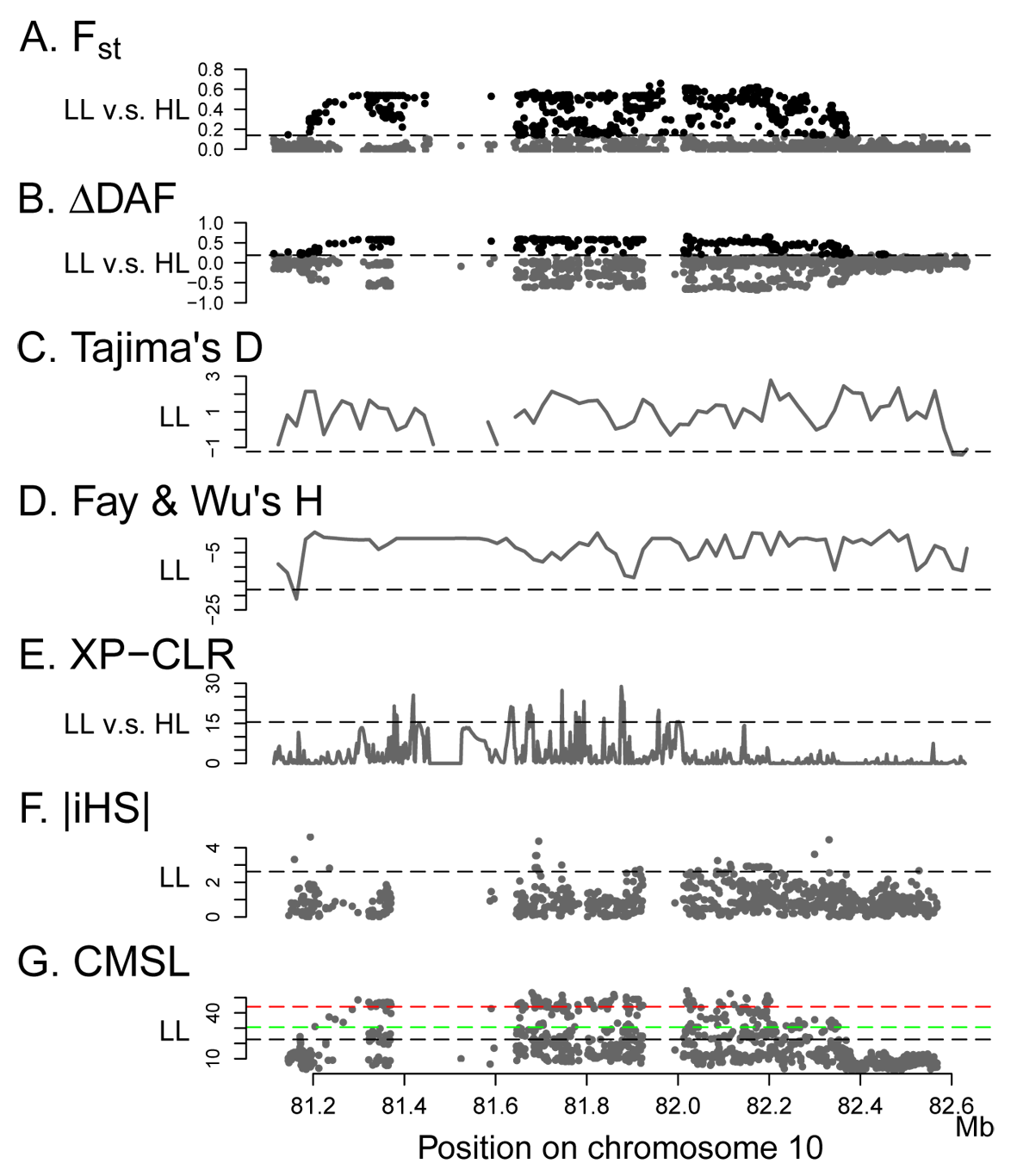

Supplement: S4 Fig — (A) F ST between HL and LL. (B) Derived allele frequencies of LL. (C) Tajima’s D of LL. (D) Fay & Wu’s H of LL. (E) XP-CLR of LL against HL. (F) Absolute iHS score of LL. (G) CMSL score of LL. Black dashed lines are the 5% threshold of each test in standard simulations, green lines are the 1% threshold, and red lines are the 0.1% threshold. (TIF) [file pone.0125444.s004.tif]

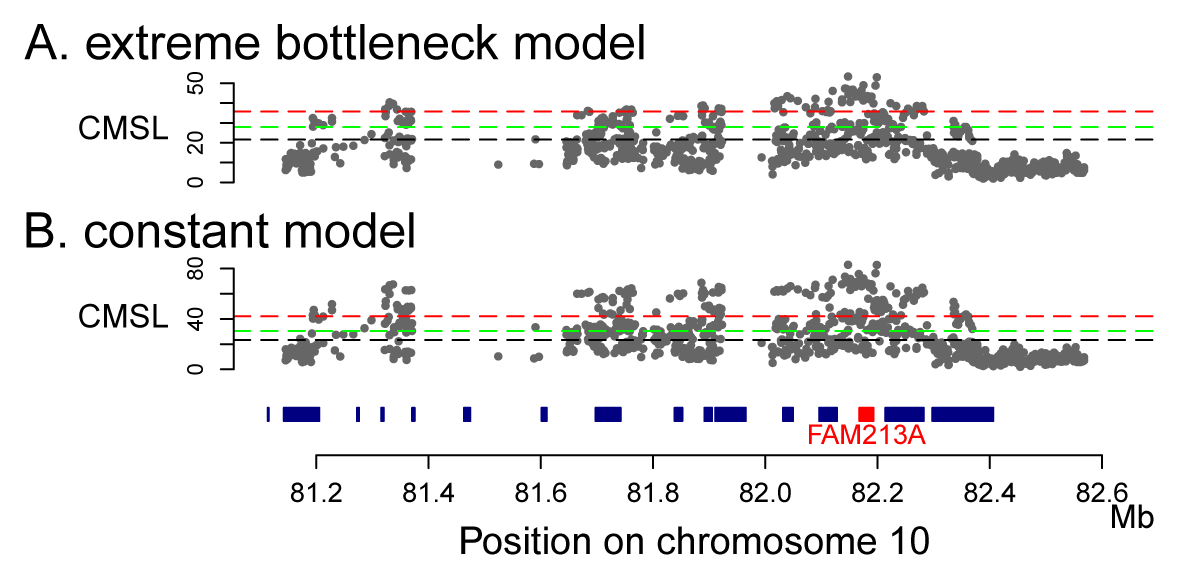

Supplement: S5 Fig — CMSL in highland populations under (A) the extreme bottleneck model and (B) the constant population size model. (TIF) [file pone.0125444.s005.tif]

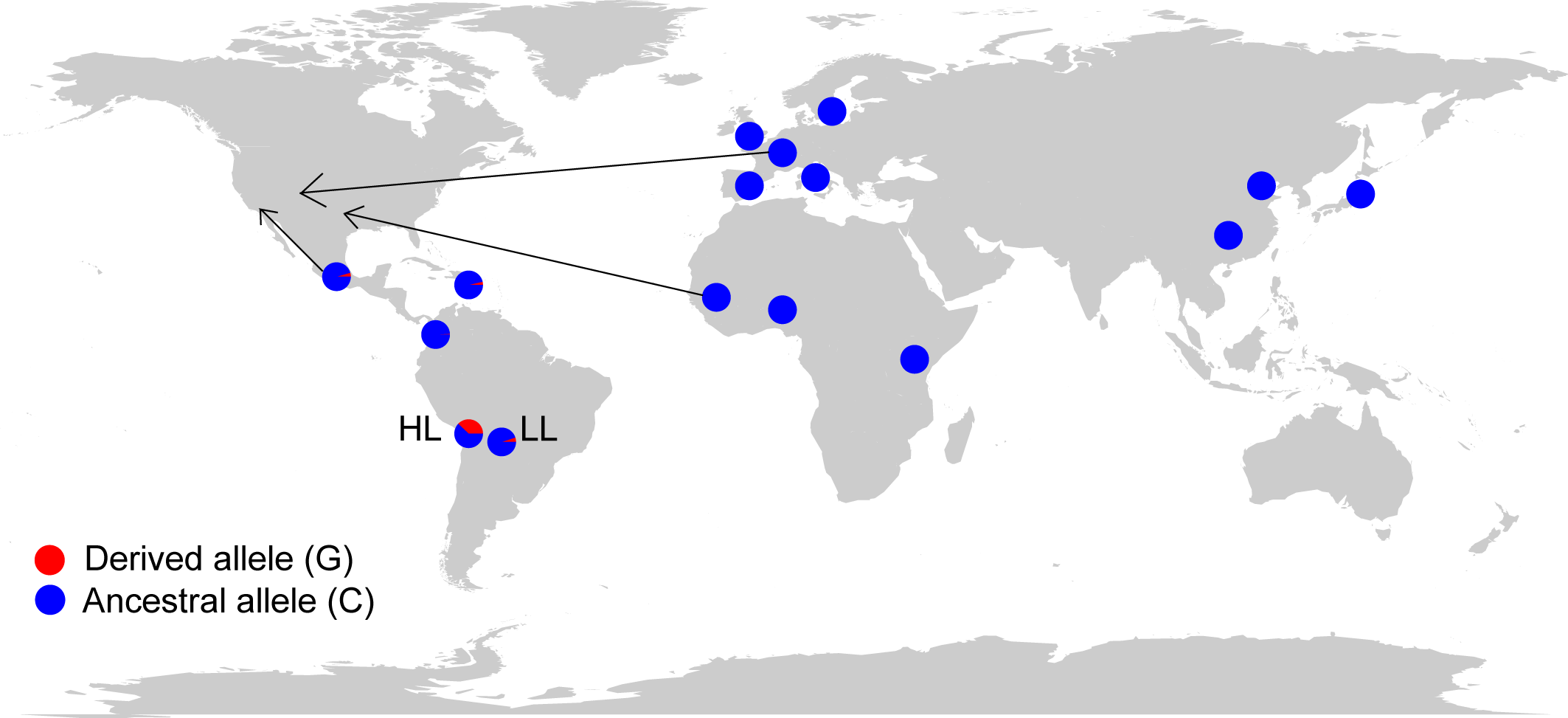

Supplement: S6 Fig — Data are from this study and 1000 Genomes. (TIF) [file pone.0125444.s006.tif]

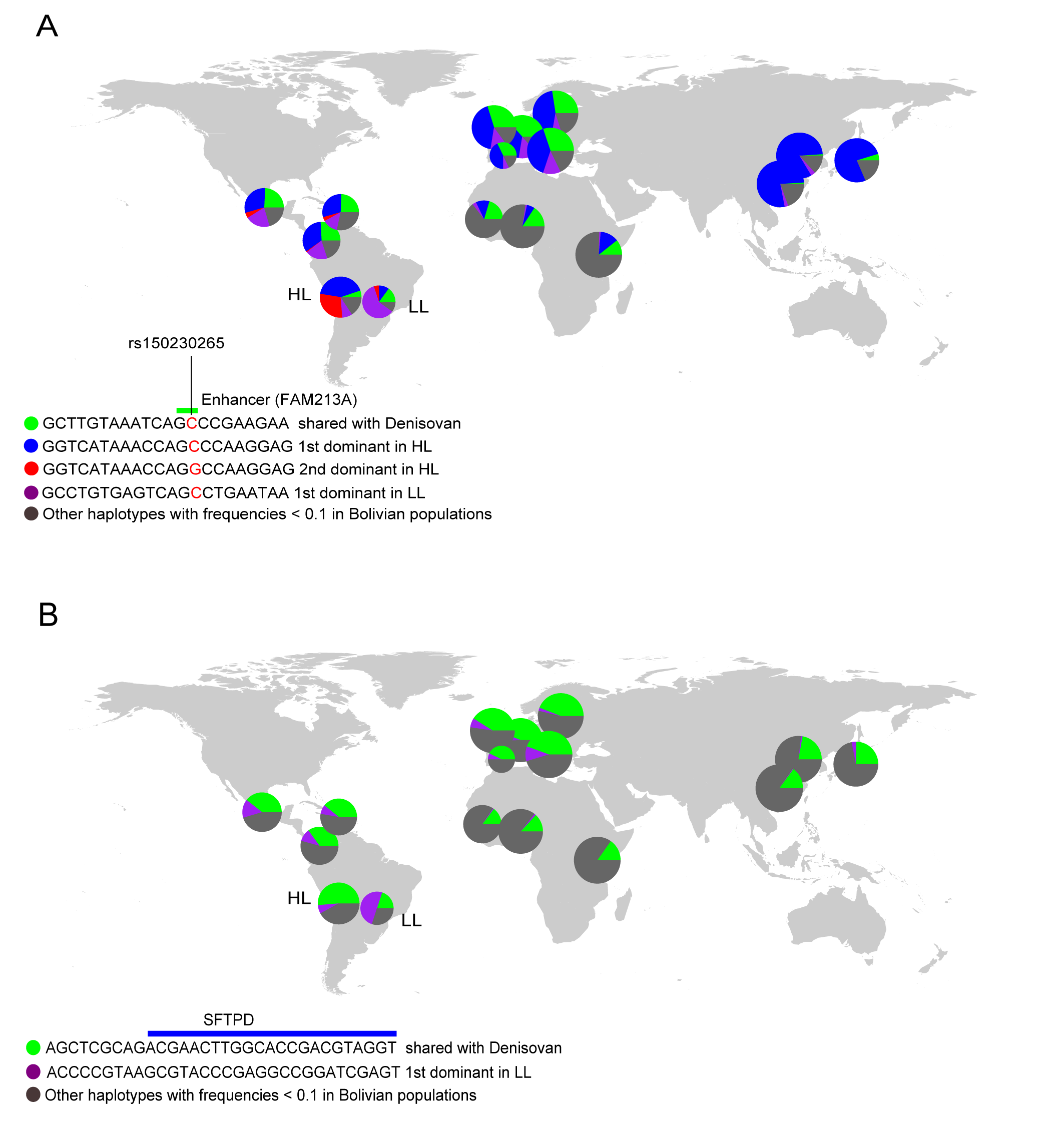

Supplement: S7 Fig — Haplotype frequencies in modern humans (from this study and 1000 Genomes data) and Denisovan genome sequence in FAM213A and SFTPD (low quality Denisovan sites were filtered following [87]). Green is haplotype shared with Denisovan; blue is the first dominant haplotype in HL; red is the second dominant haplotype which contain the derived allele of rs150230265 in HL; purple is the first dominant haplotype in LL; gray are haplotypes with frequencies <10% in Bolivian populations. The radii are scaled by sample sizes. (A) Haplotypes are 10kb extended on both sides from rs150230265. (B) Haplotypes are 10kb extended on both sites from the three non-synonymous SNPs in SFTPD. (TIF) [file pone.0125444.s007.tif]
